# Expectations for Adopting Virtual Reality to Promote Health Literacy in Patients With Persistent Pain: Qualitative Analysis of UK-Based Physiotherapists

**DOI:** 10.1155/prm/5547227

**Published:** 2025-08-29

**Authors:** Nathan Skidmore, Cormac G. Ryan, Jagjit Mankelow, Denis Martin

**Affiliations:** ^1^Centre for Rehabilitation, School of Health and Life Sciences, Teesside University, Tees Valley, Middlesbrough TS1 3BX, UK; ^2^NIHR Applied Research Collaboration for the North East and Cumbria, Cumbria, UK

**Keywords:** chronic pain, health literacy, patient education as a topic, virtual reality

## Abstract

**Background:** Persistent pain is a complex global issue, which has a significant impact on quality of life. Poor health literacy further impacts the quality of life in people with persistent pain. It is recommended that education be provided to improve health-related knowledge. VR is an engaging learning tool and could improve health literacy. Research exploring the feasibility of physiotherapists using VR to develop health literacy is minimal.

**Objectives:** To determine the feasibility of a VR-based pain education system among physiotherapists and understand barriers and facilitators to its adoption in clinical practice.

**Methods:** Semistructured interviews were conducted with physiotherapists in the United Kingdom after they used a VR-based pain education system, which combines sensory-altering experiences with pain science education. Thematic analysis was used to identify considerations related to its feasibility and its potential to influence health literacy in patients with persistent pain.

**Results:** All participants (*n* = 12) believed that the VR system could develop several aspects of health literacy, such as information understanding and appraisal. Challenges to clinical integration include allowing for increased clinical time and system training and ensuring the use of VR represents both personalized and evidence-based care.

**Conclusion:** The VR pain management system was considered a feasible adjunct to address health literacy by increasing the plausibility of information and addressing health-related understanding, appraisal, and application. Future research is required to validate the effectiveness of VR-based education systems to improve health literacy.

## 1. Introduction

Persistent pain is defined as a pain that persists or reoccurs for at least three months and is estimated to affect 20%–30% of people globally [1, 2]. Facilitating patient self-management is required to prevent and reduce the impact of persistent pain [3]. The National Institute for Health and Care Excellence (NICE) recommends that education should be delivered to patients, as part of good clinical practice, in order to develop health-related understanding [4]. Education aiming to develop patient's knowledge of the biological processes which underpin pain, in order to reduce pain and its associated disability, is known as pain science education (PSE) [5, 6] and has been shown to facilitate patients' ability to cope with their condition, increase knowledge of pain, and reduce factors influencing the pain experience including disability, kinesiophobia, and catastrophizing [7, 8].

Addressing patient health literacy, which includes not only increasing health-related knowledge but also a patient's ability to obtain, appraise, and apply health information [9], warrants attention as poor health literacy issues are prevalent in people with persistent pain and are associated with poor self-management and long-term health outcomes [10–14]. Oosterhaven and colleagues recently identified that low health literacy levels also impact the understanding and appraisal of PSE, and noted that reducing the complexity of PSE for those who have low levels of health literacy is a perceived priority among patients with persistent pain [15]. Visual aids, graphics, or pictures may provide a suitable solution and can enhance patient understanding of health-related concepts [16]. The provision of multisensory forms of information utilizing modern technologies may provide a more effective method of PSE delivery compared to conventional means.

An example of such technologies is virtual reality (VR), a computer-simulated experience, which immerses its user within a virtual environment [17]. VR has been shown to be an engaging learning tool and beneficial in promoting an understanding of health status [18] and increasing patient satisfaction, knowledge, and health-related understanding [19]. It may also provide a solution to research identifying a need to facilitate information understanding and appraisal alongside a lack of health information application associated with PSE [15].

Building on our previous study, which demonstrated the acceptability and feasibility of VR PSE among healthcare professionals in primary care settings [20], the present study extends this work by exploring the feasibility of initial adoption in clinical practice. While continuous usage has been previously examined, the role of VR within the context of initial adoption, where the tool has not yet been used, remains underexplored. This distinction is important, as the determinants of behavioral intention differ between initial adoption and continued use [21]. Feasibility of ongoing use is typically informed by experience, whereas initial adoption is shaped by broader beliefs and attitudes.

Understanding this early-stage feasibility requires further investigation. In particular, it involves assessing the characteristics of initial adoption behavior intention within clinical practice for the development of health literacy, which is crucial for determining its overall feasibility and planning of its successful implementation [22]. Low acceptance of such technologies may result in delays to, or even failure of, potentially successful systems [23], therefore identifying key drivers influencing acceptance is key. Previous research has shown that there is an appetite among physiotherapists for VR to support rehabilitation [24], and that the delivery of educational content via VR is feasible and acceptable to patients and clinicians when aiming to develop patient health knowledge [25]. Research expanding this perceived feasibility to include the delivery of VR PSE in the context of developing health literacy is lacking.

Therefore, the aims of this study were to (a) determine physiotherapists' perceptions of using a VR PSE system to develop health literacy in patients with persistent pain and to (b) understand potential barriers and facilitators to its initial adoption within clinical practice.

## 2. Methods

### 2.1. Design, Procedure, and Participants

A feasibility study exploring the opinions of physiotherapists using qualitative semistructured interviews was conducted. Physiotherapists based in the North-East of England were invited to participate in the study and were recruited via the use of email and advertisement on social media. A convenience sample of physiotherapists registered with the Healthcare Professions Council (HCPC), who practiced privately within a musculoskeletal service, were invited via direct email to participate in the study. Participants were excluded if they had experienced motion sickness from previous use of VR and/or were not willing to participate in a recorded interview. This study did not seek to achieve complete data saturation but rather information power [26] by focusing on obtaining in-depth interviews with a homogenous sample of clinicians with expertise in the field of pain education. An initial approximation (informed by consensus within the research team and that of evidence for the concept of data saturation [27]) was sought. However, emphasis was solely placed on information power, in that adequacy of the final sample size was evaluated iteratively by the research group based on how the sample specificity, theoretical background, quality of dialog, and fruitfulness of analysis are in regard to the study aims [26].

Participants who met the inclusion criteria were invited to participate in two sessions: (a) a session in which the participant used the VR system and (b) a semistructured interview. The first session included the participants using the VR PSE system for 30–45 min, in which they were encouraged to complete as many modules as possible to develop insight into the material included. The total time to complete all modules is approximately 40 min, with participants permitted to take a break at any time. Individuals were then asked to complete a 60-min interview to provide their opinions on the potential use of the system in clinical practice. Participant's written consent was obtained before the usage session, and interviews were conducted immediately afterward. To facilitate participation, options to conduct an online interview within 14 days were also provided. Sessions were conducted individually by the main investigator and took place, according to participant preference, in a consultation room at either the private clinical practice of the participant, or on the university campus.

### 2.2. VR Pain Education System

**Figure 1:**
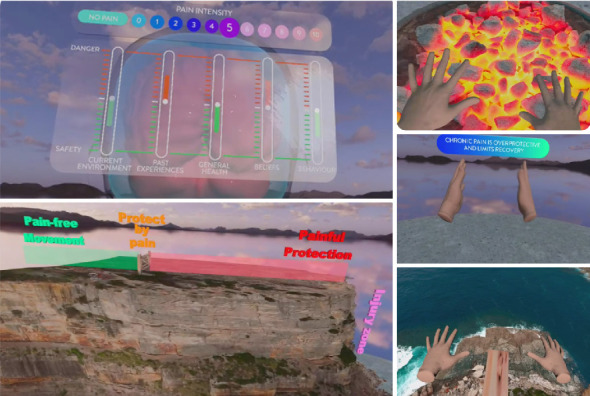
Reality Health VR Pain Education platform *(with permissions)*.

The Reality Health Pain Education Platform (Figure 1) delivered via VR is an immersive educational experience combining sensory-altering experiences with evidence-based PSE, movement, diaphragmatic breathing, mindfulness, and graded exposure therapy. Modules included in the VR system feature the following:

• Introductory module: An immersive environment to facilitate acclimatization in the VR world with an introduction to the virtual avatar and breathing exercises.
• Understanding pain: Immersive and interactive PSE focusing on the biopsychosocial perspective of pain including concepts about pain that patients have previously identified as valuable to them [28].
• Retrain your body: delivery of modern pain science to commence retraining of the pain system via interactive analogies and visualizations linked to movement and concepts of neuroplasticity.
• Retrain the brain: understanding the concept of “pain amplification” and how one's current environment, past experiences, general health, beliefs, and behaviors can influence pain system hypersensitivity.
• Rehabilitation: provides immersive light movements in a nonthreatening environment. Activities are aimed at providing graded exposure therapies while altering sensory input.

Before participants used the system, they were informed that it was designed to be delivered as an adjunct to a clinical consultation rather than independently without the involvement of a healthcare professional. This was to facilitate the development of opinions reflecting feasibility within clinical practice.

### 2.3. Data Collection and Analysis

**Figure 2:**
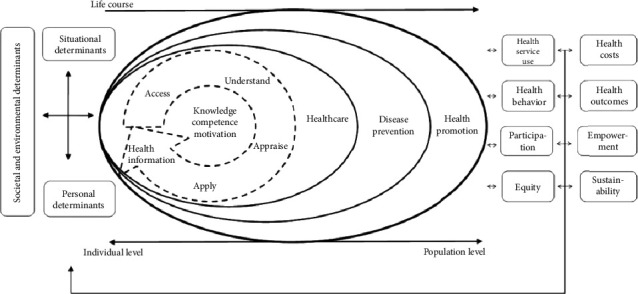
Integrated model of health literacy *(permissions: Creative Commons Attribution License)*.

To capture opinions related to feasibility and acceptability, semistructured questions were developed by the research team and informed by the unified theory of acceptance and use of technology (UTAUT-2) [29] and the integrated model of health literacy (IMHL) [9]. The UTAUT-2 examines six key constructs (performance expectancy, effort expectancy, social influence, facilitating conditions, hedonic motivation, and price value) that influence behavioral intention and technology use. The IMHL serves as a basis for developing health literacy–enhancing resources (Figure 2). The core competencies (accessing, understanding, appraising, and applying health-related information) related to the development of skills enable a person to navigate three domains of the health continuum (healthcare, disease prevention, and health promotion). Questions were developed with the aims of ascertaining opinions relating to these constructs and aimed to extract opinions of (1) the hardware, (2) the ability of the tool to develop a “health-literate” patient, and (3) the software used in the system. Main topic questions were supplemented with additional prompt (optional) questions (Supporting Information 1) to encourage further reflection on specific aspects of system usability and feasibility.

Interviews were audio-recorded, transcribed, and anonymized by substituting participant names with unique identifiers (e.g., CL01) and replacing any reference toward a participant with a neutral reference (e.g., they and them). Transcripts were then exported to software (NVivo 12) for analysis. Data analysis was conducted using thematic analysis as suggested by Clarke et al. [30]. We chose to use an inductive-essentialist approach focusing on semantic meaning within the data, as this was in keeping with the research aims whilst facilitating a pragmatic and data-driven approach. Transcripts were read by the first author (NS) and then re-read for familiarization before initial coding. As attempting to gain consensus of meaning and assessing accuracy and reliability of coding is not suggested within the scope of reflexive thematic analysis, the use of independent reviewer(s) was not required [30]. Once initial codes were created, themes were then developed and named, reflecting meaningful concepts related to intervention use, acceptability, and health literacy. Regarding trustworthiness, Guba's criteria ensured that credibility was supported through prolonged engagement and reflexivity, dependability and confirmability were enhanced via an audit trail and research team discussions, and transferability was addressed through detailed descriptions of participants and context [31].

## 3. Results

### 3.1. Overview

In total, 12 healthcare professionals participated in the study. The characteristics of participants can be found in Table 1. Eight interviews (66.7%) were conducted immediately after the participant used the VR, and the remaining four (33.3%) were conducted within 14 days via online video calls.

**Figure 3:**
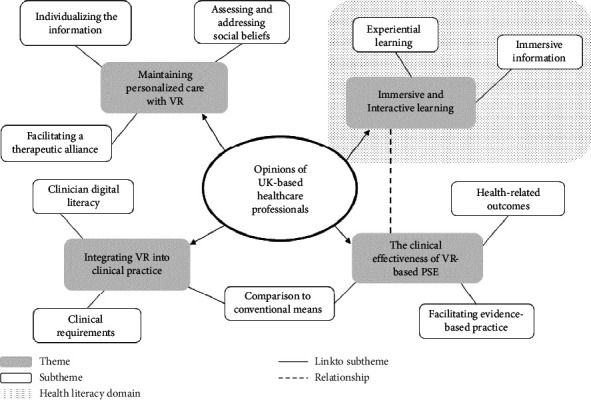
Thematic map.

A thematic map was developed to illustrate themes within the data (Figure 3). Overall, physiotherapists expressed that the VR system was deemed a feasible and potentially superior means of addressing health literacy, specifically, increasing the plausibility of information via the provision of experiential education. Themes describing opinions related to the use of the system focused on the role of immersive and interactive learning to develop health literacy, using VR to maintain personalized care, integrating VR into clinical practice, and understanding the clinical effectiveness of VR PSE.

#### 3.1.1. Theme 1: Immersive and Interactive Learning for Health Literacy

Participants believed that the capacity of the system to deliver information in an immersive and experiential way was its main attribute. This referred to the combination of an immersive 360-degree audio/visual stimulus, prioritizing the focus of attention toward interactive and practical elements of the learning content. The most common perceived benefit of this immersive form of information was that of increased information understanding and appraisal. It was suggested by participants that VR has the potential to provide superior learning outcomes compared to conventional methods such as leaflets, lectures, and websites due to users being active participants in the information.

“So people can experience (…) like their hands heating up or the hitting a balloon, which is not there and feeling like it is there. So that evidence that you could feel like a physical perception without that physical stimulus, and I think that may be used to enhance the plausibility and credibility of there being a number of factors which can influence our perception, and thus a number of factors which can influence their pain. (…) I believe that it's that experiential learning which is a very powerful facilitator. Like that old Chinese proverb: Tell me, I forget, show me, I remember, involve me, I understand. There's something about being more active and seeing it through their own eyes that makes it believable, real, understandable.” (CL08)

Participants felt VR had the potential to enhance the plausibility and credibility of information, via experiential learning, which is deemed as important in information reconceptualization as defined by Posner's four pillars of reconceptualization; (a) a dissatisfaction with existing concepts, and that new concepts must be (b) intelligible, (c) appear initially plausible, and (d) should be fruitful [32]. Participants also stated that visceral responses created by elements within the virtual experience could be powerful in providing “lived experiences” of PSE, such as how the brain reacts to dangerous situations (e.g., standing on a cliff edge).

“I think, rather than just imagine standing on a cliff and this is how you feel, they're actually experiencing it though they know they're not on the cliff, so it makes it real for them.” (CL05)

Negative comments about immersive and experiential learning were also apparent in the data, notably toward the experiences being overwhelming. It was suggested that the immersion may have a negative influence on patient's ability to focus on the didactic information delivered within the immersive experience.

“You want to maximize the learning from the exposure and your didactic style of content delivery. If you're doing both at the same time, they're just getting in each other's way. The learner is going to miss out on those key points.” (CL08)

#### 3.1.2. Theme 2: Maintaining Personalized Care With VR

Participants often highlighted the importance of providing personalized care to patients and identified potential threats associated with the provision of this type of technology. Individualizing the information, facilitating a therapeutic alliance, and assessing individual beliefs were discussion points within this theme. Comments reinforced the idea that the system could be used as an adjunct to the provision of information in a unique and experiential format, which may be identified as a preferred option after consultation with individual patients, thus facilitating personalized care.

“The individual with pain needs to be heard, felt, understood, and listened to and accepted. Then you guide them, and they want to find out more, then we have this great tool.” (CL09)

In enabling personalized care, assessment of user suitability (e.g., patient age, comorbidities, and physical restrictions) is of importance. An example related to the use of VR is that wearing a physical device on their head may be unsuitable for some patients. Participants additionally noted that in ensuring adequate user suitability and personalized care using this technology, understanding patient beliefs, familiarity, and expectations of such technology would be required. Participants expressed that despite the perceived effectiveness of the VR system, it may not be appropriate for all patients.

“There is no one size fits all. You can't just give somebody a program and say, there you are. It's got to be patient-centered.” (CL05)

#### 3.1.3. Theme 3: Integrating VR Into Clinical Practice

Ensuring that the system and its implementation into clinical practice are a feasible process was of importance to participants. It was felt that considerations related to ensuring adequate time to use the device during appointments (including setup and/or troubleshooting time) alongside physical clinical space and the provision of training were a requirement to its successful implementation.

“I think it's going to add time into an appointment. I think that's a slight issue that some people might see as a barrier.” (CL01)

“I think there's loads of value to it. I'm not sure clinical practice is ready for it, but that's more from a staffing skills confidence perspective, than the actual technology.” (CL07)

It was also noted that, in order to consolidate information and facilitate understanding, it is a requirement that the system should be used by clinicians who possess an adequate understanding of PSE, and that the system should not act as a substitute for, or be operated by, a clinician without such knowledge.

“What's important is having somebody there, whether it's a physio or somebody who's trained in pain science, to actually guide the person through it and to make sure that they understand the information.” (CL04)

#### 3.1.4. Theme 4: The Clinical Effectiveness of VR PSE

Clinical effectiveness and how the VR system provides added value over conventional means of information delivery were of interest to the participants. Ensuring robust evidence supporting the use of such tools and their capacity to directly influence outcomes related to persistent pain and health literacy was considered a requirement for the successful integration into clinical practice. It was noted that participants were unsure about the clinical effectiveness of the system, which fueled concerns regarding their professional capacity to feasibly include this approach within the provision of evidence-based care.

“You always want to know what the outcomes are. Which, at the moment, I don't know. Incorporating into clinic at the moment would be pretty difficult to do.” (CL02)

“This has to be effective. It (the system) can't just be a new selling point. It has to be clinically effective.” (CL11)

Opinions of participants highlighted that the best application of the system would be that it provides a supplementary means of health information delivery, and that it may be best used as a reflection and reinforcement tool for complex concepts related to PSE. Providing PSE in a conventional way was seen not only as a prerequisite, but also as something that should occur between encounters to help consolidate information. The VR system would then be used to consolidate learning in an immersive audio/visual format.

“I think for those patients who are engaged in pain education can start to understand it. So it would almost be like you are reinforcing what has already been said. I don't think you could just say I'm going to talk to you about persistent pain, have the VR. I think they would need to have some degree of understanding (…) and then you are reinforcing it.” (CL05)

Participants were aware of some evidence reflecting its evidence-based clinical effectiveness, noting the capacity of VR to provide relaxation and distraction experiences for patients. This provided encouragement about the capacity of VR to address health literacy and fueled the belief that VR could, at a minimum with current evidence, provide a useful means of relaxation and immersive escapism, which may be beneficial for some patients.

“As soon as you came on, you did have the bird noises. I could imagine that that would bring people into a nice, relaxed kind of state which is useful.” (CL04)

## 4. Discussion

### 4.1. Principal Findings

The aims of this study were to determine physiotherapists' opinions of the feasibility of a VR PSE system to develop health literacy in patients with persistent pain and to understand potential barriers and facilitators to its initial adoption within clinical practice. The findings suggest that a VR-based pain education system is perceived to be a potentially feasible adjunct in addressing health literacy in patients with persistent pain. Specifically, physiotherapists believed that the provision of VR-based PSE has the potential to enhance the plausibility and credibility of pain-related information, thus positively influencing information understanding, appraisal, and application. These findings align with and extend our previous research, which suggested initial enthusiasm for the use of VR-based PSE among primary care clinicians [20]. Whereas the earlier study explored acceptability in principle, the current study adds depth by examining adoption feasibility following direct system use by physiotherapists.

### 4.2. Immersive/Experiential Learning for Health Literacy

Physiotherapists believed that the immersive nature of VR contributed significantly to the novelty of this method of health information delivery. The benefits of providing immersive educational experiences are well documented in the literature [18, 19, 33, 34], which reflect the opinions expressed in this research. Physiotherapists in this study believed that providing health information in an immersive and interactive fashion facilitates the development of competencies related to health literacy such as information understanding and appraisal. This is attributed to the ability of VR to provide its users with immersive and experiential learning, which physiotherapists believe to be the key to facilitating patient understanding and appraisal of complex information related to the concepts of PSE. Explaining the influence of thoughts, emotions, and experiences on one's pain experience is a key aspect of PSE [28]. Given the need for PSE to be intelligible and plausible [15, 32] and the benefits of experiential learning to facilitate reflection, conceptualization, and behavioral change [35], our findings suggest that utilizing VR as an adjunct medium to deliver PSE has the potential to address issues related to patients experiencing difficulties with understanding messages delivered within PSE, negatively appraising information, and struggling to apply PSE information in daily life [15].

Participants also believed that the system was able to influence patient's ability to access and apply health-related information, although this was to a lesser and indirect extent. Applying health information, for example, enhancing health-related communication skills, relies on information comprehension [9]. Participants believed that the VR-based approach was able to influence information understanding and appraisal, thus affecting information comprehension. It is via these means that VR is believed to influence an individual's capacity to apply health-related information. Although research is required to quantify and validate these perceived benefits of VR as a form of PSE delivery to influence health literacy, physiotherapists believe it to be a feasible tool to support those with persistent pain in this context.

It was not believed that the system positively addressed patient access to information. Such opinions, which are reflected in previous research, show that the current accessibility of VR in healthcare is relatively low [36]; therefore, it was believed that, with regard to accessing information, this system provided little benefit over cheaper and more freely available forms of PSE (such as leaflets and websites). This may change as the development, affordability, and clinical relevance for the use of VR within healthcare advance.

### 4.3. Providing Personalized Care Through VR

Ensuring personalized care is of great importance to the management of persistent pain [4]. Our results suggest that, similar to previous research, VR is believed to pose a potential risk to the development of a collaborative approach to care (e.g. a therapeutic alliance), attributed partly to its lack of face-to-face interactions [37]. Although delivered in a face-to-face setting, concerns about whether patients perceive the treatment as patient-centered, attributed to apprehension associated with the provision of care for which an intervention is delivered in a standardized format, may negatively influence patient expectations, thus posing a threat to clinical outcomes. Exploration of whether these beliefs are reciprocated in the opinions of patients will be important; however, our findings suggest that clinicians show concerns about the potentially detrimental effects of VR on the development of a therapeutic relationship and the delivery of personalized care.

### 4.4. Integrating VR Into Clinical Practice

VR is considered a feasible means of information delivery within healthcare [38, 39]. Findings within this study expand on previous work, highlighting the continuous usage intention by providing insight into the delivery of PSE to support the management of persistent pain within the context of initial adoption. Despite this, there are numerous points of consideration for healthcare professionals in order to efficiently integrate VR into clinical practice. To ensure the delivery of personalized care and to provide a crucial opportunity for the clinician to ascertain levels of patient understanding and appraisal and to assess health-related outcomes, physiotherapists suggest that VR PSE should be supplemented with healthcare professional–led discussions with patients outside of the virtual experience. Therefore, an essential component of the delivery of VR PSE is that the healthcare professional possesses a comprehensive understanding of the concepts related to PSE. Also in line with previous research [20, 40], results from this study show that physiotherapists believed that one must consider the time required to set up, deliver, and troubleshoot such technologies and their ease-of-use in comparison to conventional means. Other considerations include financial implications (specifically in relation to the provision of information via conventional means, often with minimal cost) and the clinical space required to conduct interactive elements of VR. Recommendations to tackle the obstacles to the initial adoption of this approach include employing a cooperative strategy for its integration into clinical practice, emphasizing its potential compensability, ensuring adequate staff training, and considering the time constraints of using VR PSE within a private setting. Findings from this research not only highlight factors which influence the feasibility of VR PSE but also indicate a requirement for future research to formally and systematically explore the complex role VR PSE has on influencing a patient's ability to understand, appraise, and apply health-related information and examine its clinical utility.

### 4.5. Strengths and Limitations

This study builds upon prior research by incorporating a broader range of factors related to the feasibility and application of VR in clinical practice. Utilizing inductive data analysis methods, this research extends beyond previous deductive approaches to offer a comprehensive perspective. This study explored the opinions of privately practicing physiotherapists in the North-East of England; thus, the findings may not transfer to other settings. It is also noted that recruitment may have been biased towards individuals with a particular interest in this technology and topic. While interview prompts were employed with flexibility to facilitate reflection on key topics, certain prompts may be considered inherently directive and were not applied uniformly across all interviews. This inconsistency may have introduced response variation or potential bias, including acquiescence bias, which should be taken into account when interpreting the findings [41]. Although methods used in this study are sufficient in addressing the aims of this research, future research may benefit from exploring the feasibility of this system during its use in clinical practice.

## 5. Conclusion

This study explored the opinions of physiotherapists on a VR-based PSE system to address health literacy in patients with persistent pain. Physiotherapists consider a VR PSE system to be a feasible adjunct to developing health literacy in patients with persistent pain, and it is potentially advantageous in increasing the plausibility and credibility of information related to PSE. The initial adoption feasibility of such an intervention is perceived to rely on its cost, social perceptions, patient suitability, effectiveness to support health-related outcomes, and capacity to facilitate personalized care. Future research investigating the clinical utility of VR PSE systems is required to verify these opinions and to further explore the unique potential for VR to enhance the plausibility and credibility of PSE.

## Figures and Tables

**Table 1:** Participant characteristics.

Participant characteristics		
Age (years)	Mean (range)	43.5 (25–64)
Gender	Female	2
	Male	10

Length of qualification (years)	Mean (range)	17.3 (2–34)
Used VR in clinical practice before?	Yes	0
	No	12

## Data Availability

Additional information can be found in the supporting information.

## Supporting Information

Supporting 1: Semistructured interview questions.
