# Supplementary material for: Expectations for Adopting Virtual Reality to Promote Health Literacy in Patients With Persistent Pain: Qualitative Analysis of UK-Based Physiotherapists
Source: Pain Res Manag. 2025 Aug 29;2025:5547227. doi: 10.1155/prm/5547227 (PMC12413278; doi:10.1155/prm/5547227)
Supplement: Supporting Information — Additional supporting information can be found online in the Supporting Information section. [file 5547227.f1.pdf]

Supplementary Materials

Supplementary 1: Semi-structured interview questions.

<table>
<tr><th>Topic</th><th>Question</th></tr>
<tr><td colspan="2">Part 1: The Hardware (Aim: 1,2)</td></tr>
<tr><td>Virtual Reality (VR) format</td><td>What are your thoughts about using virtual reality within the clinical setting?
Additional Prompt Questions:
Have you used VR before?
What do you think about using the VR?
What are some good points about using VR?
What are some bad points about using VR?
Was the VR easy to use?
How do you think this might feel to a patient to use?
Have you thought about using VR in your practice before? If so/not, why?
Could the usage of VR fit into your daily practice?
Do you feel like VR hardware would be a good investment for practice?
How do you think VR could be used in clinical practice?
What considerations might a clinic need to be able to effectively include VR as a treatment tool?</td></tr>
<tr><td colspan="2">Part 2: Virtual Reality to develop a 'health-literate' patient (Aim: 3)</td></tr>
<tr><td colspan="2">The interviewer will then provide the following statement providing a working definition of health literacy to provide context to the following questions. The statement reads as follows: "Definitions of Health Literacy are yet to be confirmed with consensus within the research community. However, Health Literacy can be defined as the knowledge, motivation and ability for patients to access, understand, appraise and apply health related information".</td></tr>
<tr><td>Health Literacy for the patient</td><td>What does health literacy mean to you?
What are your thoughts about the role of developing health literacy in patients with persistent pain?</td></tr>
</table>

	Additional Prompt Questions:<br>• Why do you think/ not think that health literacy is important?<br>• Are there any other elements of health literacy you don't understand?<br>• Do you believe that VR could have a role in developing pain-related health literacy?<br>• In what areas of health literacy do you think VR could be effective?<br>• Do you think VR may be able to address a patient's ability to access good health information in their day-to-day life?<br>• Do you think VR could be a good tool to addresses a person's ability to understand health-related information in their day-to-day life?<br>• Do you think VR is able to address a person's ability to appraise and evaluate health-related information?<br>• Do you think VR could support a person's ability to use health-related information in their day-to-day life?<br>• Do you think there are any other areas of a patient's management that this tool could address?<br>• Are you /have you experienced any difficulties when addressing a patients health literacy? Could VR be a solution to these difficulties?
Part 3: The Software *(Aim: 1,2)*	
Content of the software	• **What do you think about the content delivered in the VR experience?**<br><br>*Additional Prompt Questions:*<br>• What do you think about the information delivered in the VR?<br>• Did you find the information understandable?

<table>
<tr><td></td><td>Was there any information which did not make sense?
Is this information similar to what you might deliver in clinic?
What do you think a patient might think about the information being delivered?
If any, what sort of content is missing from the experience?</td></tr>
<tr><td>Immersion</td><td>Did you find yourself immersed in the experience? If so/not, why?
Additional Prompt Questions:
Did you find yourself immersed in the experience?
How did you find navigating the experience?
What are your thoughts about how the experience looks?
Do you think making a patient feel immersed is important?
How do you think a patient would feel immersed?</td></tr>
<tr><td>Interactivity</td><td>Did you find the experience interactive? If so/not, why?
Additional Prompt Questions:
How did you find the interactive elements of the experience?
How important is it for an experience to be interactive for a patient?</td></tr>
</table>
